# Direct-to-Satellite IoT Slotted Aloha Systems with Multiple Satellites and Unequal Erasure Probabilities

**DOI:** 10.3390/s21217099

**Published:** 2021-10-26

**Authors:** Felipe Augusto Tondo, Samuel Montejo-Sánchez, Marcelo Eduardo Pellenz, Sandra Céspedes, Richard Demo Souza

**Affiliations:** 1 Department of Electrical and Electronics Engineering, Federal University of Santa Catarina, Florianópolis 88040-900, Brazil; felipe.tondo@posgrad.ufsc.br (F.A.T.); richard.demo@ufsc.br (R.D.S.); 2 Programa Institucional de Fomento a la Investigación, Desarrollo e Innovación, Universidad Tecnológica Metropolitana, Santiago 8940577, Chile; 3 PPGIa-Graduate Program in Computer Science, Pontifical Catholic University of Parana, Curitiba 80215-901, Brazil; marcelo@ppgia.pucpr.br; 4 NIC Chile Research Labs and Department of Electrical Engineering, Universidad de Chile, Santiago 8370451, Chile; scespedes@ing.uchile.cl

**Keywords:** machine-type communications, IoT, satellite communications

## Abstract

Direct-to-satellite Internet of Things (IoT) solutions have attracted a lot of attention from industry and academia recently, as promising alternatives for large scale coverage of a massive number of IoT devices. In this work, we considered that a cluster of IoT devices was under the coverage of a constellation of low-Earth orbit (LEO) satellites, while slotted Aloha was used as a medium access control technique. Then, we analyzed the throughput and packet loss rate while considering potentially different erasure probabilities at each of the visible satellites within the constellation. We show that different combinations of erasure probabilities at the LEO satellites and the IoT traffic load can lead to considerable differences in the system’s performance. Next, we introduce an intelligent traffic load distribution (ITLD) strategy, which, by choosing between a non-uniform allocation and the uniform traffic load distribution, guarantees a high overall system throughput, by allocating more appropriate amounts of traffic load at different positions (i.e., different sets of erasure probabilities) of the LEO constellation with respect to the IoT cluster. Finally, the results show that ITLD, a mechanism with low implementation complexity, allows the system to be much more scalable, intelligently exploiting the potential of the different positions of the satellite constellation.

## 1. Introduction

Terrestrial low-power wide area networks (LPWANs) are already massively deployed, enabling several applications that short-range or cellular communications technologies cannot support in an efficient way [1,2]. New technologies such as LoRaWAN [3] and SigFox [4] are extensively utilized all over the world in a number of different use cases. However, there has been increased interest in enabling truly global connectivity for Internet of Things (IoT) devices, which can only be achieved with satellite communications. Global, or very wide-area, IoT connectivity can leverage several applications to their full potential, in areas such as environmental monitoring, disaster prevention, smart agriculture and industrial digitalization [5,6,7,8]. Global IoT can be used to map possible environmental disasters such as earthquakes and floods, and to provide connectivity to under-served regions. In particular, it is expected that low-Earth orbit (LEO) satellites will play a fundamental role in this scenario, providing direct or indirect satellite communication to a massive number of devices [9,10].

The space industry has been growing exponentially in recent years due to remarkable advances in the manufacturing of satellites and rocket launching technologies, and the development of embedded systems circuits and communication technologies [11]. One of the attractive outcomes under these successive evolutions has been a significant reduction in the cost of communication via LEO satellites. With its relatively low cost compared to geostationary-Earth orbit (GEO) satellites, modular implementation and low latency, this type of satellite has gained considerable attraction for future applications. In this context, LEO orbit satellites would be beneficial due to their smaller propagation signal loss and potential global coverage. Many private sector players are investing massively in the launching of small satellites, as SpaceX [12] and OneWeb [13]. LoRaWAN technology is already being used in IoT networks assisted by LEO satellites—for instance, those of Lacuna Space (https://lacuna.space/ (accessed on 14 September 2021)) and Swarm Space (https://swarm.space/ (accessed on 14 September 2021)), in which most traffic is from the devices to the LEO satellites. Another example of a successful IoT system using LEO satellites is the ARGOS system (https://www.argos-system.org/ (accessed on 14 September 2021)). Moreover, there are ongoing standardization efforts in order to integrate satellites with 5G terrestrial networks, with IoT being one of the use cases [14]. In line with the fact that one LEO is not enough to provide service to a large IoT network, a strong trend for applications in future networks is the deployment of several satellites spread across space, that is, a constellation [15].

### 1.1. State-of-the-Art

In the design of a satellite IoT network, several factors are of paramount importance, directly influencing the system performance, such as constellation design, number of satellites, number of orbital planes, elevation angle, orbital plane spacing, and the orbital eccentricity [16]. Added to these factors is the wireless access technique used by the IoT devices. Different methods have been proposed to establish a connection between a device and a satellite, with their own advantages and disadvantages. We start with indirect communication, in which data are transmitted to the satellite via a terrestrial gateway. Therefore, the biggest advantage is the possibility to use current terrestrial LPWAN technologies at the end nodes. The IoT devices actually connect to the terrestrial gateway, in which protocols, such as LoRaWAN and SigFox, can be used to connect to end nodes [1]. A disadvantage of this architecture is the limited terrestrial coverage [17]. Another negative factor is the difficulty of installing a gateway in remote regions or in cases where monitoring is carried out for a short period of time, which makes the cost of the service higher.

Arguably, the most attractive method in terms of ease of deployment is direct satellite communication, better known as direct-to-satellite (DtS-IoT) [5]. In this case, users communicate directly to the satellite, most probably a LEO satellite, without the need for a terrestrial gateway. However, some of the challenges of this approach are the long link distance and the short communication windows. LEO satellites are concentrated at altitudes between 340 and 650 km [6] and provide time windows for communication according to their passing orbits. Some manufacturers in the field of wireless communications [3,4] have been working on cutting-edge technologies that are getting closer and closer to meeting the challenge of DtS-IoT. In [17], interesting advances in the interconnection of technologies were already presented to allow adequate DtS-IoT communication design using a satellite constellation and LPWAN technology such as LoRaWAN.

In addition to direct and indirect communication, other alternatives are also presented, such as the hybrid land-satellite network (HSTN) proposed in [18]. Due to characteristics such as communication delay, latency, mobility, and coverage performance, the authors proposed three basic cooperative models for the HSTN. In the so-called *L* model, the satellite communicates with the device via a terrestrial gateway (i.e., indirect communication). An example of a network operating in the *L* model with LoRaWAN terrestrial devices can be found in [19]. In the *X* model, devices communicate using direct and indirect techniques. Communication between users-satellite and users-terrestrial base station is executed separately, sharing wireless resources. Interference patterns found in this model are diverse and complicated, such as mutual interference between users. Furthermore, they are dependent on channel fading influences and cell coverage effects. In urban areas, the satellite user and the terrestrial user coexist, yielding unwanted co-channel signals. The *V* model considers the cooperation of a satellite with a terrestrial base station to serve the same user, being therefore a combination of the indirect and direct approaches. Signal diversity reception is used to compensate for large channel fading. Furthermore, the terrestrial and satellite network protocols may be different, requiring translation.

Independent on the architecture, whether direct, indirect, or a combination of both, medium access control (MAC) techniques implemented in commercial satellite networks were not designed to provide scalable solutions for the growing number of devices envisioned for IoT. Traditional solutions such as Code Division Multiple Access (CDMA) and Time Division Multiple Access (TDMA), when placed in the context of LEO satellites and large device density, may lose performance due to the need for strict synchronization. Requirements such as simplicity, storage, and energy consumption should be incorporated into the designs of MAC protocols for satellite IoT networks [20]. With a focus on DtS-IoT networks, a taxonomy of MAC protocols is presented in [20], including four groups: (i) Aloha-based; (ii) reservation and adaptive protocols; (iii) interference cancellation based; and (iv) hybrid protocols. A detailed analysis on the trade-offs involving complexity and scalability is provided. The authors finished their review with the conclusion that a better balance among different metrics should drive the design of novel MAC protocols for DtS-IoT networks. In particular, it is apparent that the choice of a MAC protocol for satellite IoT networks should carefully consider the complexity of implementation and energy consumption.

Therefore, random access (RA) protocols based on Aloha [21] are good candidates for the MAC layer in LEO satellite IoT networks, both in terms of simplicity of implementation and delay [20]. Indeed, they have been used for satellite communications for a long time and are even used in modern terrestrial networks, such as LoRaWAN [3] and SigFox [4], and are becoming attractive alternatives for some future 6G use cases [22]. However, for a large number of transmitters, the system’s performance can be severely affected by collisions. One solutions for this issue is the introduction of diversity in Aloha. Modern RA schemes [23] based on Aloha often apply SIC for interference cancellation, while allowing devices to transmit multiple copies of their messages [24,25,26,27,28]. For instance, in Contention Resolution Diversity Slotted Aloha (CRDSA), devices transmit fixed numbers of replicas of their messages while successive interference cancellation (SIC) is applied at the receiver for removing all copies once one of the messages is successfully decoded, considerably improving performance [26]. In Irregular Repetition Slotted Aloha (IRSA), devices may transmit different numbers of replicas, improving even more the network throughput [27]. An application of IRSA in the context of satellite communications, where the number of replications per user is optimized, is exploited in [28].

However, methods such as CRDSA and IRSA produce time diversity by means of replication, which may lead to considerable increases in complexity and power consumption at the transmitters, while also demanding substantially in terms of memory and computational complexity at the receiver for the SIC operations. Therefore, in the context of LEO satellites with limited computational resources, time diversity techniques such as CRDSA and IRSA may still be prohibitive. Another alternative is the use of spatial diversity at the receiver [29,30]. Nowadays, even the use of massive Multiple Input Multiple Output (MIMO) antenna systems at the receiver has been considered in the context of machine-type communications using Aloha [31,32]. Although promising, a small LEO satellite with several receive antennas may still not be a very practical option.

Spatial diversity can also be exploited with Aloha by having multiple single antenna receivers [33,34]. This idea fits well in a scenario where several satellites cover a given region, which should be more and more common with the predicted launch of hundreds of satellites in the next years [35]. For instance, the links between devices and the multiple relays (e.g., LEO satellites) in coverage have been modeled considering on–off fading (i.e., erasure probabilities at the satellites) in [15,36], where different analysis and optimizations were carried out. In [15] critical and non-critical devices coexisted in a slotted Aloha based communication system with multiple satellites. Then, the satellites forwarded the received data to a sink over another shared backhaul link. Orthogonal and non-orthogonal strategies for allocating resources among critical and non-critical services were investigated. The multiple relays can be seen as a constellation of LEO satellites, and the common sink can be an Earth station.

Recently, in [36], a two-phase communication system was proposed and analyzed. The system model in [36] is similar to that in [15], so that in the first phase, a group of clustered devices transmit their packets to multiple relays or multiple satellites using a simple slotted Aloha protocol. Then, in the second phase the relays forward the decoded information to a common sink. The channel is modeled considering on–off fading, while expressions are provided for calculating the first phase throughput, in addition to the packet loss rate for a number of relays, considering equal erasure probabilities at all relays. However, in the case of a LEO satellite constellation, it is very likely that the erasure probabilities are not all the same, since some of the satellites may be at different elevation angles from the point of view of the devices. The same is true even for other alternative constellation designs, as the GeoSurf constellation [37], since satellites entering or leaving the service are subject to an additional loss compared to the satellite currently servicing the devices.

### 1.2. Novelty and Contribution

In this work, we build on the model proposed in [36], with a focus on the first phase, in which the devices communicate with the satellites. However, differently from [36], here we consider that the erasure probabilities at the different satellites are not necessarily the same. Then, we reformulate the throughput and the packet loss rate to take into account the different erasure probabilities at the satellites. Next, using the novel throughput formulation, we exploit non-uniform traffic load distribution as a function of the number and position (which implicates in the erasure probabilities) of the multiple satellites. We show that adequately allocating the traffic intensity according to the number and positions of the satellites brings considerable benefits, and for that sake the modeling proposed in this work is fundamental.

The rest of this work is organized as follows. In Section 2 we present the system model. In Section 3 we derive expressions for the throughput and packet loss rate considering different erasure probabilities at the satellites. An algorithm for traffic load optimization is introduced in Section 4. Numerical results are discussed in Section 5, and Section 6 concludes the paper.

## 2. System Model

We consider that a very large number of clustered devices are under the coverage of *K* LEO satellites, then, following the related literature, we focus on the uplink, where most of the data traffic is concentrated and where contention can be very high. Devices transmit data packets to the satellites following a simple slotted Aloha policy [20]. Although this is out of the scope of this work, the LEO satellites can forward the data to an Earth station in a following phase. Moreover, we assume that there is minimum, if any, coordination among satellites, so that they do not exchange information among them regarding the received messages. The channel between the devices and the satellites is modelled considering on–off fading [36,38], so that the satellites perceive a given erasure probability $\varepsilon_{k}$, k∈{1,2,...,K}. The erasure probabilities $\varepsilon_{1},\varepsilon_{2},...,\varepsilon_{K}$ depend on the position of the satellite with respect to the cluster of IoT devices. Note that the on–off fading model has been extensively used in the analysis of Aloha-based multiple access schemes, as for instance in [15,23,25,33,36]. Such model describes well the behavior of channels whose losses are mainly dominated by fading and short-term blocking due to the presence of obstacles [33,36], as in the case of IoT applications supported by end-devices transferring data via LEO satellites. Moreover, the on–off fading model brings the bonus of mathematical tractability, without loss of generality.

We use the term position to refer to the continuous segment within the satellite’s trajectory where the erasure probability does not vary significantly, while we call iteration the time it takes for a LEO satellite to transit from one position to the next. Then, the positions associated with a satellite constellation with respect to a cluster of IoT devices are the set of all those in which at least one satellite of said constellation is visible to the cluster. Thus, the first position is that in which the first satellite is seen, and the last position is the one in which the last satellite is seen before disappearing from the cluster range. Moreover, every passing of the satellite constellation over the IoT cluster is termed a lap.

Since the satellites may be in different positions with respect to the IoT cluster, it is natural to assume that the erasure probabilities may be different at the different satellites. For the sake of simplicity, we assume that there is a finite number *n* of different erasure probabilities experienced by the satellites, such that $\varepsilon_{k}\in \left\{\varepsilon_{1},\varepsilon_{2},\cdots ,\varepsilon_{n}\right\}$. Moreover, also for the sake of simplicity, we assume that every segment associated with the different positions has the same length and then all iterations are of equal duration. Each iteration is associated with a fixed angular variation Δθ, such that the number of positions located around the Earth is equal to $\frac{360^{\circ }}{\Delta \theta }$. Finally, note that the lowest erasure probability happens at the position associated with the zenith, and the highest erasure probabilities are associated with positions close to the horizon.

Unlike [36], where all satellites perceive the same erasure probability (which would only happen if orbiting together or very close to each other), in this paper we exploit a model where the erasure probability depends on the satellite’s orbital position with respect to the cluster of IoT devices. In order to determine the impact of the satellite constellation design on the system performance, we consider different topologies, given according to a fixed *spacing* $s\in \left\{0,1,2\cdots 9\right\}$, in number of positions, between consecutive satellites.

**Example** **1.** *Table 1 lists the positions and the erasure probabilities in a topology with K=2 satellites, when either s=0 (both satellites orbit together) or s=1 (both satellites are separated by a single position; i.e., in the next iteration the second satellite occupies the current position of the first satellite). Moreover, we consider a set of n=10 different erasure probabilities, $\varepsilon \in \left\{0.01,0.1,0.2,0.3,0.4,0.5,0.6,0.7,0.8,0.9\right\}$. These erasure probabilities could be biunivocally related to the elevation angle of the satellite with respect to the cluster, from the zenith ($90^{\circ }$) to the horizon (close to $0^{\circ }$) with intervals of $\Delta \theta =10^{\circ }$, as the severity of fading and shadowing is a function of the elevation angle [39,40,41]. Note that with s=0 there are in total 19 positions, and when s=1 there is one more position with at least one satellite being visible by the IoT cluster.*

It is easy to check that in the general case the number of positions in each lap is given by

(1) M=2n−1+(K−1)s.

Note that this interpretation of *M* is valid as long as $M\leq \frac{360^{\circ }}{\Delta \theta }$, but in constellations with too many satellites or where the satellites are too far apart from each other, such that $M>\frac{360^{\circ }}{\Delta \theta }$, *M* still coincides with the number of iterations associated with each lap of the satellite constellation.

**Figure 1:**
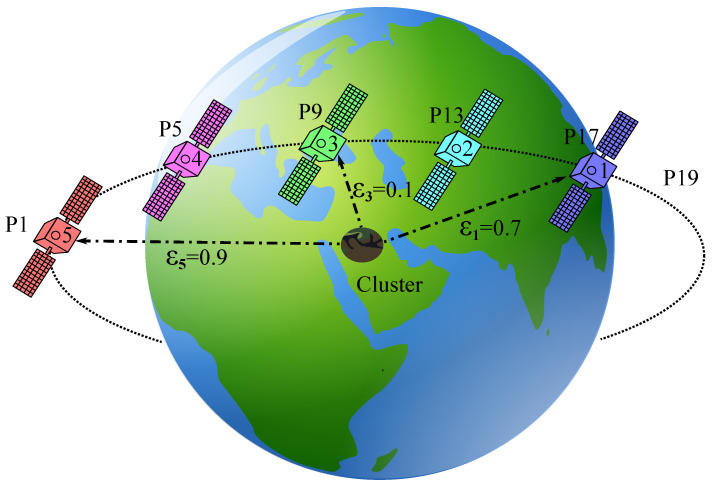
System model topology: K=5 satellites with different erasure probabilities ε for a spacing of s=4 positions between consecutive satellites. The snapshot corresponds to the 17th position of $Sat_{1}$.

**Example** **2.** *To facilitate the understanding of the system model, we present in Figure 1 a snapshot of the 17th positions of the first satellite in a constellation of K=5 satellites, with a fixed satellite spacing of s=4 positions, and assuming that ε∈{0.01, 0.1, 0.2, 0.3, 0.4, 0.5, 0.6, 0.7, 0.8, 0.9}. Some aspects of interest that should be noted: (i) each satellite only has 19 visible positions from the point of view of the cluster and experiences the erasure probability values in decreasing order $\varepsilon_{1}\in \left\{0.9,0.8,\cdots ,0.01\right\}$ until it is just above the cluster, where it experiences the smallest value $\varepsilon_{1}=0.01$, and from this point on it begins to experience the erasure probability values in increasing order $\varepsilon_{1}\in \left\{0.01,0.1,\cdots ,0.9\right\}$; (ii) however, this topology has M=35—that is, when the first satellite is still visible to the cluster in another 16 iterations, some of the remaining satellites are visible, the first being only visible in the 19 first positions and the last satellite only being visible in the last 19 positions; (iii) this means that when s increases, the number of iterations in which a satellite is visible to the cluster increases, but at the same time the number of positions in which the K satellites of the constellation are visible decreases; (iv) even for some topologies it would not be possible to have the K satellites in the same iteration (e.g., K=4 and s=9)—in such circumstances the non-visible satellites can be considered in the analysis with $\varepsilon_{k}=1$.*

Moreover, in the considered model neither retransmission policies nor communication between satellites are considered. Having in mind that low cost satellites may have limited computational capabilities, it is also assumed that collisions are destructive; i.e., we do not consider multiuser detection or successive interference cancellation at the LEO satellites. Following [36], the average number of packets transmitted per slot is defined as *G*, and the number of users accessing the channel at the same time-slot is modeled as a Poisson random variable *U*, so that the probability that *u* users transmit in the same time-slot is

(2) $$P\left[U=u\right]=\frac{G^{u}e^{-G}}{u!}.$$

## 3. System Throughput and Packet Loss Rate

In this section we derive expressions for the system throughput and the packet loss rate considering the system model in Section 2.

### 3.1. Throughput

A packet is only successfully received at the *k*th satellite if it has not been erased by the channel fading realization and if there is no collision of other non-erased packets transmitted by other users at the same time-slot. Thus, given that *u* packets were simultaneously transmitted by different users in a given time slot, the event of a successful packet reception at the *k*th satellite occurs with probability $q_{k}=u\left(1-\varepsilon_{k}\right)\varepsilon_{k}^{u-1}$. Then, the throughput, defined as the number of packets successfully received per time slot, at the *k*th satellite, is:

(3) $$\begin{array}{cc} T_{k}\left(G\right) & =\sum_{u=0}^{\infty }q_{k}\;P\left[U=u\right]\\ \; & =\sum_{u=0}^{\infty }\left[u\left(1-\varepsilon_{k}\right)\varepsilon_{k}^{\left(u-1\right)}\frac{G^{u}e^{-G}}{u!}\right]\\ \; & =G\left(1-\varepsilon_{k}\right)e^{-G\left(1-\varepsilon_{k}\right)}. \end{array}$$

The system throughput (T) is defined as the number of packets successfully received by at least one of the satellites per time slot. Therefore, multiplicities must be discarded, so that the system throughput is not only the sum of the throughput experienced by each of the satellites. For that sake, the inclusion–exclusion principle [42] can be utilized to determine the cardinality of the union of the sets of packets successfully received by each satellite, thereby discounting for the intersections.

For instance, for the case of three sets $D_{1}$, $D_{2}$, and $D_{3}$ (in our case each set would contain the data packets successfully received by a different satellite) via the principle of the inclusion-exclusion we can determine the number of different received packets as: $|D_{1}\cup D_{2}\cup D_{3}\left|=\right|D_{1}\left|+\right|D_{2}\left|+\right|D_{3}\left|-\right|D_{1}\cap D_{2}\left|-\right|D_{1}\cap D_{3}\left|-\right|D_{2}\cap D_{3}\left|+\right|D_{1}\cap D_{2}\cap D_{3}|$. The elements within the $D_{1}$, $D_{2}$, and $D_{3}$ sets that are double-counted are removed. This can be generalized to the case of *K* sets as [42,43]

(4) $$\left|\overset{K}{\underset{k=1}{⋃}}D_{k}\right|=\sum_{J\neq \emptyset ,\;J\subseteq \{1,\ldots ,K\}}{\left(-1\right)}^{|J|+1}\left|\underset{j\in J}{⋂}D_{j}\right|,$$

where J is a set with indexes of the subsets whose intersection must be evaluated. Note that the above expression does not admit a simplification step applied in [36], which requires the setup to be symmetrical. In our particular case, as the erasure probabilities are different, the ordering (or numbering) of the satellites is relevant.

Then, it is fundamental to know $\left|{⋂}_{j\in J}D_{j}\right|$, the cardinality of the intersection of the sets of packets successfully received by a subset J⊆{1,…,K} of satellites with cardinality |J|. Consequently, considering the traffic model described in Section 2, given that *u* packets were simultaneously transmitted by different users in a given time slot, the event that the same packet is successfully received by the |J| satellites occurs with probability

(5) $$q_{J}=u\prod_{k\in J}\left(1-\varepsilon_{k}\right)\varepsilon_{k}^{\left(u-1\right)}.$$

After the realization of many time-slots *N*, the average number of packets jointly received by |J| satellites, for all *u*, is $\left|{⋂}_{j\in J}D_{j}\right|=N\sum_{u=0}^{\infty }q_{J}P\left[U=u\right]$. Thus, the system throughput T(G), with different erasure probabilities at the satellites, is

(6) $$\begin{array}{cc} T(G) & =\lim_{N\to \infty }\frac{1}{N}\sum_{J\neq \emptyset ,\;J\subseteq \{1,\ldots ,K\}}{\left(-1\right)}^{|J|+1}\left|\underset{j\in J}{⋂}D_{j}\right|\\ \; & =\sum_{J\neq \emptyset ,\;J\subseteq \{1,\ldots ,K\}}{\left(-1\right)}^{|J|+1}\sum_{u=0}^{\infty }\left[\frac{G^{u}e^{-G}}{u!}u\prod_{k\in J}\left(1-\varepsilon_{k}\right)\varepsilon_{k}^{u-1}\right]\\ \; & =\sum_{J\neq \emptyset ,\;J\subseteq \{1,\ldots ,K\}}{\left(-1\right)}^{|J|+1}e^{-G}\prod_{k\in J}\left(1-\varepsilon_{k}\right)\sum_{u=0}^{\infty }\left[\frac{uG^{u}}{u!}{\left(\prod_{k\in J}\varepsilon_{k}\right)}^{u-1}\right]\\ \; & =\sum_{J\neq \emptyset ,\;J\subseteq \{1,\ldots ,K\}}{\left(-1\right)}^{|J|+1}\;G\;\prod_{k\in J}\left(1-\varepsilon_{k}\right)\;\;e^{-G\left(1-\prod_{k\in J}\varepsilon_{k}\right)}. \end{array}$$

For instance, in the particular and practical case of K=2, we have that J=$\left\{\{1\},\{2\},\{1,2\}\right\}$ and $T_{2}\left(G\right)=G\left(1-\varepsilon_{1}\right)e^{-G\left(1-\varepsilon_{1}\right)}+G\left(1-\varepsilon_{2}\right)e^{-G\left(1-\varepsilon_{2}\right)}-G\left(1-\varepsilon_{1}\right)\left(1-\varepsilon_{2}\right)e^{-G\left(1-\varepsilon_{1}\varepsilon_{2}\right)}$, but when $\varepsilon_{1}=\varepsilon_{2}=\varepsilon$ the system throughput becomes $T_{2}\left(G\right)=2G\left(1-\varepsilon \right)e^{-G\left(1-\varepsilon \right)}-G{\left(1-\varepsilon \right)}^{2}e^{-G\left(1-\varepsilon^{2}\right)}$, the same as in ([36], Equation (6)). Finally, it is important to remark that in the case of equal erasure probabilities, $\varepsilon_{k}=\varepsilon$, ∀k, the cardinality of the union in (4) becomes ∑k=1KKk(−1)k−1⋂j∈JDj as in ([36], Equation (5)). Then, after some manipulations, the throughput in (6) can be written as in ([36], Equation (2)), and therefore the model of equal erasure probabilities is a particular case of the formulation presented in this subsection.

### 3.2. Packet Loss Rate

A packet is lost if none of the *K* satellites are able to correctly receive it. In order to estimate the packet loss rate, we assume that a target user always transmits, and then we determine the probability that its packets cannot be successfully decoded.

Assuming that *u* packets were simultaneously transmitted by other users in the same given time slot, the event of the successful reception of the target packet (from the target user) at the *k*th satellite occurs with probability $p_{k}=\left(1-\varepsilon_{k}\right)\varepsilon_{k}^{u}$. Then, the packet loss rate, i.e., the packet loss probability from the target user, at the *k*th satellite, is:

(7) $$\begin{array}{cc} P_{k} & =1-\sum_{u=0}^{\infty }p_{k}\;P\left[U=u\right]\\ \; & =1-\sum_{u=0}^{\infty }\left[\left(1-\varepsilon_{k}\right)\varepsilon_{k}^{u}\frac{G^{u}e^{-G}}{u!}\right]\\ \; & =1-\left(1-\varepsilon_{k}\right)e^{-G\left(1-\varepsilon_{k}\right)}. \end{array}$$

From (3) and (7), we can verify that $P_{k}\left(G\right)=1-\frac{T_{k}\left(G\right)}{G}$, which is consistent with the fact that the maximum throughput achievable by a user (target user) is 1 and the maximum traffic load generated by a single user is one packet per time slot.

Then, the event that specifically the target packet is successfully received by |J| satellites, of the subset J⊆{1,…,K}, occurs with probability

(8) $$p_{J}=\prod_{k\in J}\left(1-\varepsilon_{k}\right)\varepsilon_{k}^{u}.$$

After the realization of many time-slots *N*, which implies the transmission of *N* packets from the target user, the average number of target packets jointly received by |J| satellites, for all *u*, is $\left|{⋂}_{j\in J}D_{j}\right|=N\sum_{u=0}^{\infty }p_{J}P\left[U=u\right]$. Thus, the system packet loss rate P(G) is

(9) $$\begin{array}{cc} P(G) & =\lim_{N\to \infty }\frac{1}{N}\left[N-\sum_{J\neq \emptyset ,\;J\subseteq \{1,\ldots ,K\}}{\left(-1\right)}^{|J|+1}\left|\underset{j\in J}{⋂}D_{j}\right|\right]\\ \; & =1-\sum_{J\neq \emptyset ,\;J\subseteq \{1,\ldots ,K\}}{\left(-1\right)}^{|J|+1}\sum_{u=0}^{\infty }\left[\frac{G^{u}e^{-G}}{u!}\prod_{k\in J}\left(1-\varepsilon_{k}\right)\varepsilon_{k}^{u}\right]\\ \; & =1-\sum_{J\neq \emptyset ,\;J\subseteq \{1,\ldots ,K\}}{\left(-1\right)}^{|J|+1}e^{-G}\prod_{k\in J}\left(1-\varepsilon_{k}\right)\sum_{u=0}^{\infty }\left[\frac{G^{u}}{u!}{\left(\prod_{k\in J}\varepsilon_{k}\right)}^{u}\right]\\ \; & =1-\sum_{J\neq \emptyset ,\;J\subseteq \{1,\ldots ,K\}}{\left(-1\right)}^{|J|+1}\;\prod_{k\in J}\left(1-\varepsilon_{k}\right)\;\;e^{-G\left(1-\prod_{k\in J}\varepsilon_{k}\right)}. \end{array}$$

Now, from (6) and (9) we can verify that $P\left(G\right)=1-\frac{T(G)}{G}$. Again, we give an example for the case of K=2,

(10) $$\begin{array}{cc} P_{2}\left(G\right) & =\sum_{u=0}^{\infty }\left[\frac{G^{u}e^{-G}}{u!}\right]\left[1-\left(1-\varepsilon_{1}\right)\varepsilon_{1}^{u}\right]\left[1-\left(1-\varepsilon_{2}\right)\varepsilon_{2}^{u}\right]\\ \; & =e^{-G}\sum_{u=0}^{\infty }\left[\frac{G^{u}}{u!}\right]\left[1-\left(1-\varepsilon_{1}\right)\varepsilon_{1}^{u}-\left(1-\varepsilon_{2}\right)\varepsilon_{2}^{u}+\left(1-\varepsilon_{1}\right)\left(1-\varepsilon_{2}\right){\left(\varepsilon_{1}\varepsilon_{2}\right)}^{u}\right]\\ \; & =1-\left(1-\varepsilon_{1}\right)e^{-G\left(1-\varepsilon_{1}\right)}-\left(1-\varepsilon_{2}\right)e^{-G\left(1-\varepsilon_{2}\right)}+\left(1-\varepsilon_{1}\right)\left(1-\varepsilon_{2}\right)e^{-G\left(1-\varepsilon_{1}\varepsilon_{2}\right)}. \end{array}$$

Notice that, similarly to the previous subsection when $\varepsilon_{1}=\varepsilon_{2}=\varepsilon$, the system packet loss rate becomes in $P_{2}\left(G\right)=1-2\left(1-\varepsilon \right)e^{-G\left(1-\varepsilon \right)}+{\left(1-\varepsilon \right)}^{2}e^{-G\left(1-\varepsilon^{2}\right)}$, exactly the result of ([36], Equation (7)) for K=2, showing that the model of equal erasure probabilities is a particular case of the formulation presented in this subsection too.

## 4. Intelligent Traffic Load Distribution

From the analysis in Section 3, we can conclude that the system throughput and the packet loss rate depend on the traffic load offered by the IoT devices and on the uplink erasure probabilities at the satellites. Moreover, from the system model definitions in Section 2, we know that the erasure probabilities at the satellites in each iteration depend on the position of the satellites within the constellation. Consequently, we can deduce that the optimal traffic loads (in terms of system throughput) for each position of the satellite constellation are not the same. Based on this fact, we can predict that a non-uniform traffic load distribution should outperform uniform traffic load distribution in terms of the average system throughput. Note that the cluster of IoT devices can only communicate while some of the satellites within the constellation are visible to the cluster.

Assuming a uniform load distribution, the total traffic load, $G_{T}$, to be offered during a complete lap of the satellite constellation, considering (1), can be written as

(11) $$G_{i}^{\left(u\right)}=\frac{G_{T}}{M},$$

where $G_{i}^{\left(u\right)}$, i∈{1,...,M}, is the traffic load uniformly distributed per position.

With the uniform load distribution in (11), considering both fixed total traffic load $G_{T}$ and number of satellites in the constellation, topologies with larger numbers of positions *M* allocate lower traffic loads in each position; and topologies with less positions have to allocate a higher traffic load to each position. However, such a uniform distribution does not take into consideration the location of the satellites, nor the number of satellites visible to the cluster at each position in the satellite constellation, and therefore limits performance, as in some positions the offered load may be too optimistic or too pessimistic depending on the particular erasure probabilities.

In order to increase the average system throughput, we propose an intelligent traffic load distribution (ITLD) strategy, where the system is aware of the overall throughput achievable by the uniform distribution and by a given non-uniform distribution for that total traffic load. In this proposed non-uniform distribution the traffic load offered in each position of the satellite constellation is proportional to the normalized system throughput that would be achieved in the same position with uniform load distribution. The total traffic load in a complete lap is the sum of the traffic load of all the IoT devices. So, in order to achieve a specific traffic load distribution according to the constellation positions, we estimate the load factor $Q_{i}$ in each position *i* of the satellite constellation as

(12) $$Q_{i}=\frac{T_{i}\left(G_{i}^{\left(u\right)}\right)}{\sum_{i=1}^{M}T_{i}\left(G_{i}^{\left(u\right)}\right)},$$

which represents in what proportion each position of the satellite constellation contributes to the overall system throughput, when the traffic load is uniformly distributed. Following the hypothesis that higher performing positions can successfully take on higher traffic load, we propose to use this load factor as the appropriate weight to conveniently distribute the traffic load in a simple and effective manner. Then, the non-uniform traffic load, per position, is defined as follows:

(13) $$G_{i}^{\left(nu\right)}=Q_{i}\;G_{T}.$$

Finally, to establish a fair comparison between both traffic load distribution strategies, we use the overall system throughput, which is computed as

(14) $$T^{\left(x\right)}=\sum_{i=1}^{M}\;T_{i}\left(G_{i}^{\left(x\right)}\right),$$

where x={u,nu} in the case of uniform or non-uniform distribution strategy, respectively. The implementation and comparison of both uniform and non-uniform distributions are described in Algorithm 1. The erasure probabilities of all the satellite positions visible from the IoT devices cluster are assumed to be known, along with the simultaneous positions visited by the *K* satellites of the constellation. At the end of the second lap of the satellite constellation, ITLD selects the traffic load distribution that allows to achieve the highest throughput:

(15) $$G_{i}^{\left(ITLD\right)}=\left\{\begin{array}{cc} G_{i}^{\left(nu\right)} & \mathrm{if}\;T^{\left(nu\right)}>T^{\left(u\right)}\\ G_{i}^{\left(u\right)} & \mathrm{if}\;T^{\left(nu\right)}<T^{\left(u\right)} \end{array}\right.$$

Consequently, ITLD throughput is

(16) $$T^{\left(ITLD\right)}=\max\left(T^{\left(u\right)},T^{\left(nu\right)}\right).$$

Note that this intelligent distribution does not guarantee the maximum overall system throughput per lap. However, the proposed strategy is a feasible and a simple solution to increase the overall system throughput, considering the implementation limitations of typical IoT device hardware, and the energy consumption constraints associated with the on-board satellite signal processing.
**Algorithm 1:** ITLD—intelligent traffic load distribution.1: According to the satellite spacing *s*, obtain the number of positions *M* using (1);2: Evaluating (11), compute the uniform traffic load distribution per position $G_{i}^{\left(u\right)}$;3: For $G_{i}^{\left(u\right)}$, calculate the throughput per position $T_{i}$ with (6);4: Determine the overall throughput with uniform load distribution, $T^{\left(u\right)}=\sum_{i=1}^{M}T_{i}\left(G_{i}^{\left(u\right)}\right)$;5: Find the load factor $Q_{i}$ in each position using (12);6: Following (13), compute the non-uniform traffic load distribution per position $G_{i}^{\left(nu\right)}$;7: Repeat step *4* now to find $T_{i}$ with $G_{i}^{\left(nu\right)}$ for the proposed strategy;8: Determine the overall throughput with non-uniform load distribution $T^{\left(nu\right)}=\sum_{i=1}^{M}T_{i}\left(G_{i}^{\left(nu\right)}\right)$;9: If $T_{i}^{\left(nu\right)}>T_{i}^{\left(u\right)}$, then $G_{i}=G_{i}^{\left(nu\right)}$. Else, $G_{i}=G_{i}^{\left(u\right)}$. Thus, $T=\max(T^{\left(nu\right)},T^{\left(u\right)})$.

## 5. Numerical Results

In this section we present results for the throughput and the packet loss rate, using the formulation derived in Section 3, while considering the model given in Section 2. Moreover, we also investigate the performance of the proposed traffic load distribution algorithm introduced in Section 4.

### 5.1. Throughput

**Figure 2:**
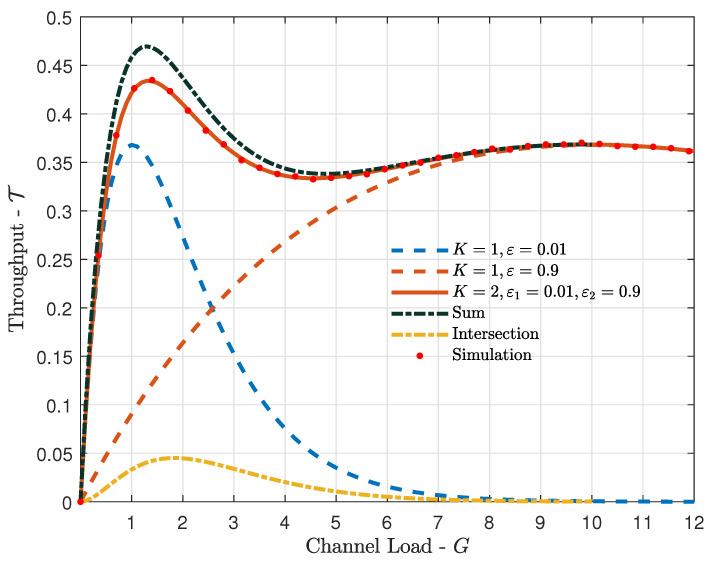
Throughput T vs. channel load *G*, for K=1 with erasure probabilities ε∈{0.01,0.9} and K=2 with erasure probabilities $\varepsilon_{1}=0.01$ and $\varepsilon_{1}=0.9$ (analytical and simulation results). Moreover, we show the sum and intersection of the individual throughputs seen at each satellite.

Figure 2 shows the throughput versus the channel load, for the cases of a single satellite with different erasure probabilities (ε=0.01 and ε=0.9), and for the cases of K=2 satellites with erasure probabilities $\varepsilon_{1}=0.01$ and $\varepsilon_{2}=0.9$. Moreover, in the figure we also show the sums of the individual throughputs seen by each of the K=2 satellites, as well as the intersections of their throughputs. Recall that the actual throughputs are the differences between the sums of the individual throughputs and their intersections (recall the inclusion-exclusion principle discussed in Section 3). First, considering the case of a single satellite, we can see from Figure 2 that a low erasure probability leads to a larger throughput at low channel loads, and a higher erasure probability is favorable at high channel loads. That is because a high erasure probability limits the collisions, which is desirable at high loads. However, a high erasure probability also leads to an inefficient utilization of the resources of the IoT devices. Interestingly, when we considered K=2 satellites, one with a low and another with a high erasure probability, the benefits were quite large, especially at low to moderate channel loads. Moreover, in the figure we also show simulation results for the case of K=2, validating the analysis in Section 3. Finally, the curves representing the sums and the intersections of the individual throughputs of the two satellites highlight the importance of removing multiplicities when deriving the throughput in Section 3, as the intersection is non-negligible.

**Figure 3:**
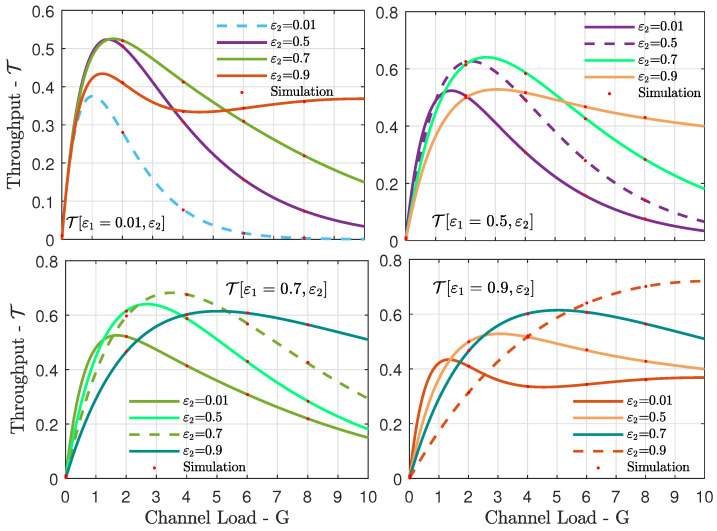
Throughput T vs. channel load *G* for the case of K=2 satellites. We fix the erasure probability $\varepsilon_{1}$ at the first satellite at a different value for each of the subfigures, and we consider a set of different erasure probabilities $\varepsilon_{2}$ at the second satellite in each subfigure.

We extended the analysis for the case of K=2 satellites in Figure 3, in which we fixed the erasure probability $\varepsilon_{1}$ at the first satellite for each of the subfigures, and we considered different erasure probabilities $\varepsilon_{2}$ at the second satellite. Thus, we could evaluate the system throughput for a large number of $\left[\varepsilon_{1},\varepsilon_{2}\right]$ pairs. Several interesting conclusions can be drawn from these results. First, having low erasure probabilities at both satellites is good only at very low channel loads. Moderate erasure probabilities at one of the satellites, or even at both, are beneficial at low to high channel loads, as they limit the impact of packet collisions. Another important outcome from the results in Figure 3 is that there is an optimum $\left[\varepsilon_{1},\varepsilon_{2}\right]$ pair for a given channel load. Finally, it is noteworthy that simulation results agree very well with the theoretical analysis in Section 3.

**Figure 4:**
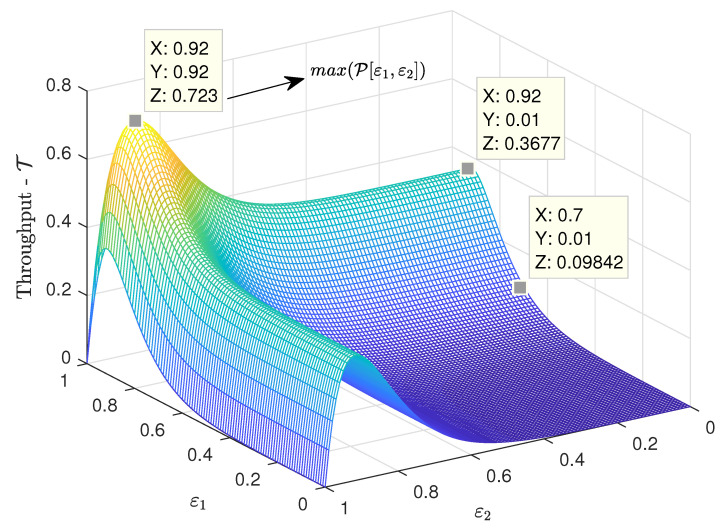
Throughput surface versus $\varepsilon_{1}$, $\varepsilon_{2}$, for the case of K=2 satellites and a high channel load of G=12 packets per time slot.

Figure 4 shows the throughput surface as a function of the erasure probabilities $\varepsilon_{1}$ and $\varepsilon_{2}$ at the satellites, for the case of G=12 packets per time slot. First, corroborating one of the conclusions from Figure 3, there was an optimum pair of erasure probabilities that led to the maximum throughput. Since in this example the channel load was very high, the maximum throughput was obtained when the erasure probabilities were also very high ($\varepsilon_{1}=\varepsilon_{2}=0.92$). However, note that such high erasure probabilities, although favorable in terms of throughput at such high channel load, also led to large packet loss rates. The relation of the packet loss rate with the channel load and the erasure probabilities is discussed in more detail in the next subsection.

### 5.2. Packet Loss Rate

**Figure 5:**
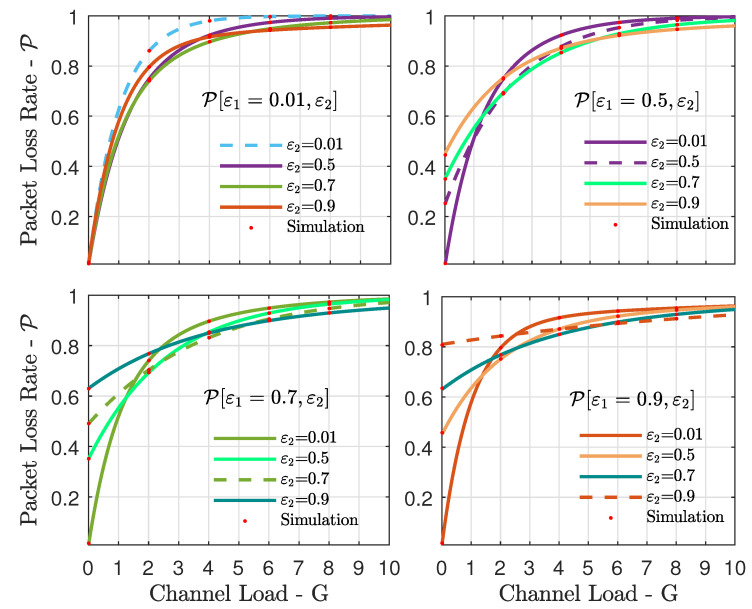
Packet loss rate P vs. channel load *G* for the case of K=2 satellites. We fixed the erasure probability $\varepsilon_{1}$ at the first satellite to a different value for each of the subfigures, and we consider a set of different erasure probabilities $\varepsilon_{2}$ at the second satellite in each subfigure.

**Figure 6:**
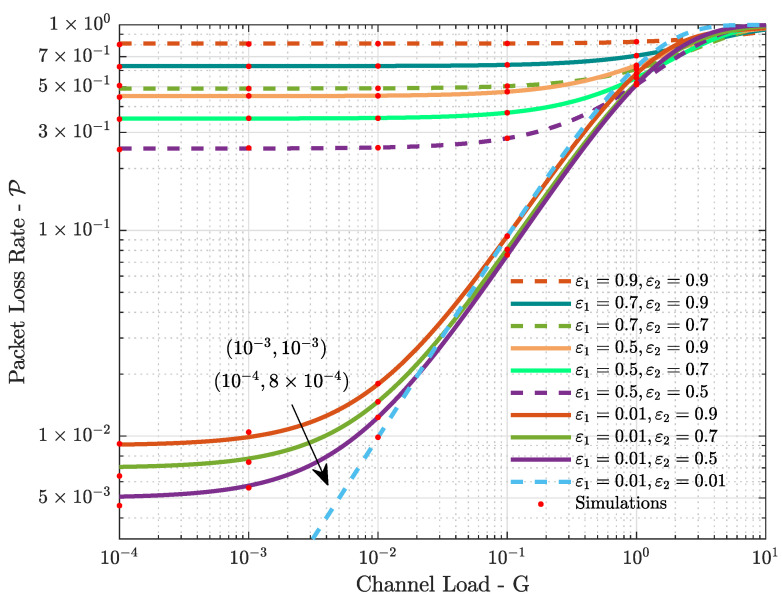
Packet Loss Rate P vs. channel load *G* for K=2 satellites and different pairs of erasure probabilities $\left[\varepsilon_{1},\varepsilon_{2}\right]$, in the case of low channel load.

In Figure 5 we investigate the packet loss rate for different pairs of erasure probabilities $\left[\varepsilon_{1},\varepsilon_{2}\right]$ in the case of K=2 satellites. In each subfigure, similarly to what was done for the throughput in Figure 3, we fixed the erasure probability $\varepsilon_{1}$ at the first satellite, while we considered different erasure probabilities $\varepsilon_{2}$ at the second satellite. Pairs of low erasure probabilities led to low packet loss rates only in the case of relatively low channel load, and in the case of high channel loads, any pair of erasure probabilities led to a high packet loss rate. Moreover, it is very interesting to analyze Figure 5 in conjunction with Figure 3. Note that pairs of erasure probability that led to high throughput in the case of high channel load, such as $\left[\varepsilon_{1}=0.01,\varepsilon_{2}=0.9\right]$ in Figure 3, led to high packet loss rates. Therefore, it is important to note that high erasure probabilities, although favorable in terms of throughput at high channel loads, may lead to inefficient usage of the available resources. Another look at the packet loss rate as a function of the erasure probabilities can be seen in Figure 6, where we assumed a low channel load. In this case, the error floor effect of the on–off fading is apparent—i.e., the erasure probabilities. Very low packet loss rates are only achievable with very low erasure probabilities and reduced channel loads.

### 5.3. Traffic Load Distribution

**Figure 7:**
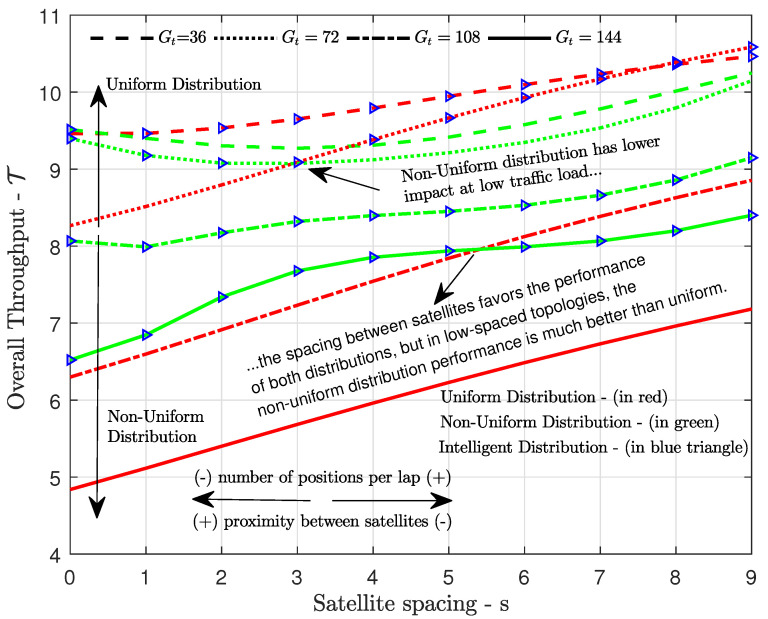
Overall throughput achieved by uniform and non-uniform traffic load distributions versus the spacing *s* between K=2 satellites, with total traffic load of $G_{T}\in \left\{36,72,108,144\right\}$ packets per lap.

Figure 7 shows the overall throughput in (14) versus the satellite spacing *s* for different total channel loads $G_{T}$, considering both the uniform and the non-uniform traffic load distributions in (11) and (13), respectively. As we can see, the non-uniform always outperformed the uniform distribution when the channel load was high, but the uniform distribution led to a larger throughput for all or several satellite spacings when the channel load was relatively low. The non-uniform distribution performed better in high channel loads because, as the traffic load increased, it ensured that the number of potential simultaneous transmissions was greater (less) in positions with greater (less) throughput. Uniform traffic load distribution became very competitive in the case of very low total traffic load, as in this case there was an excess of resources for attending such small load, which allows us to conclude that the uniform distribution limits the system’s scalability. Figure 7 also shows that, for both uniform and non-uniform distributions, the topologies with greater spacing *s* between satellites tended to achieve better performances, since they could distribute the traffic load among a greater number of positions *M*, thereby reducing the collision probability. On the other hand, in those concentrated topologies (where the satellites are very close to each other or even practically together) with large channel loads, the benefit of using non-uniform instead of uniform distribution is greater, since the number of positions where both satellites are visible is larger in these topologies, and therefore the influence of load allocation increases. All in all, since non-uniform and uniform load distribution are not the best option in every case, the proposed ITLD algorithm is able to leverage the benefits of both.

**Figure 8:**
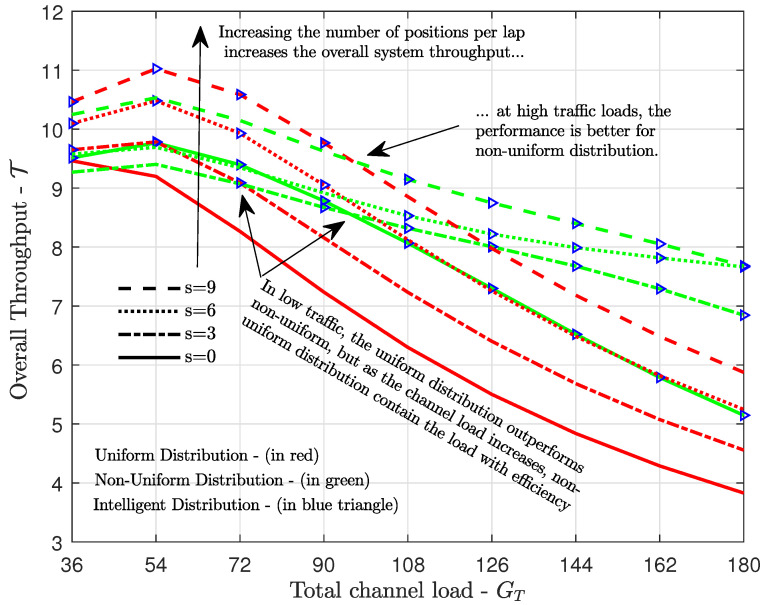
Overall throughput achieved with uniform and non-uniform traffic load distributions versus the total channel load $G_{T}$ for different spacing between K=2 satellites.

Another look at the performance of the proposed intelligent traffic load distribution method proposed in Section 4, and detailed in Algorithm 1, is given in Figure 8, which shows the throughput versus the total channel load for different spacings between K=2 satellites. Again, in the case of low channel load, the uniform distribution outperformed the non-uniform (except when both satellites are together), but with the increase of the channel load we had the opposite (for all satellite spacing considered), showing again the impact of choosing the best load distribution in each case. Moreover, we can see that in the case of smaller spacings between satellites, the advantage of non-uniform distribution tended to be greater. Once more, we can see the importance of the proposed ITLD method, which allocated the traffic load according to the system condition (total channel load, satellite spacing, and positions).

**Figure 9:**
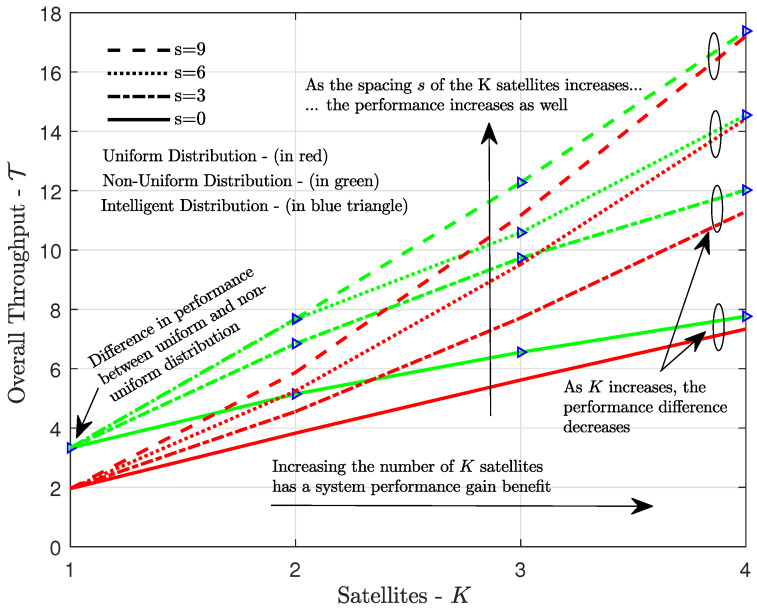
Overall throughput achieved with uniform and non-uniform traffic load distribution $G_{T}=180$ versus *K* satellites in the system.

Finally, Figure 9 shows the throughput versus the number of satellites in the constellation for a relatively high channel load and different spacings between consecutive satellites. The non-uniform load distribution clearly outperformed the uniform one for all satellite spacings, but this advantage decreased with the number of satellites. Moreover, for the two traffic load distributions under study, the throughput increased with the increase in the satellite spacing, as this generated more visible positions, allowing the system to better allocate the total channel load, thereby reducing collisions.

## 6. Conclusions

In this work we analyzed the throughput and the packet loss rate when multiple satellites cover a cluster of devices, in a direct-to-satellite IoT network. The uplink channel was modeled considering on–off fading, with potentially different erasure probabilities at each satellite. Then, we proposed an intelligent traffic load distribution algorithm, which assigns non-uniform traffic loads per position of the satellites within the constellation lap. Numerical results demonstrate that both the throughput and the packet loss rate vary considerably with the set of erasure probabilities, confirming the importance of the proposed mathematical analysis. Moreover, while also demonstrating the convenience of ITLD in terms of performance and algorithmic simplicity, the impact of the proposed non-uniform traffic load distribution was also assessed, where it became clear that in cases of high total channel load, the advantage of non-uniform distribution over the uniform one is quite large. In addition, we showed the positive impact of increasing the number of satellites within the constellation and the spacing between them.

As future works, we intend to investigate the optimum overall throughput in the case of a constellation with different sets of erasure probabilities and satellite spacing. Moreover, we also intend to design a simple (low-complexity) traffic load allocation algorithm that may approach the performance of the proposed ITLD method or even the optimum overall throughput, while adhering to the low computational and memory capabilities of IoT devices and nanosatellites. Finally, another focus will be on the exploitation of inter satellite links for providing a high level of coordination and cooperation among the satellites.

## Figures and Tables

**Table 1:** Erasure probabilities at each position (P1, P2, P3, …, P20) considering a constellation with K=2 LEO satellites ($Sat_{1}$, $Sat_{2}$), where the satellite spacing is either s=0 or s=1, and the erasure probabilities ε∈{0.01, 0.1, 0.2, 0.3, 0.4, 0.5, 0.6, 0.7, 0.8, 0.9}.

*s*	SAT	P1	P2	P3	P4	P5	P6	P7	P8	P9	P10	P11	P12	⋯	P18	P19	P20
0	$Sat_{1}$	0.9	0.8	0.7	0.6	0.5	0.4	0.3	0.2	0.1	0.01	0.1	0.2	⋯	0.8	0.9	x
	$Sat_{2}$	0.9	0.8	0.7	0.6	0.5	0.4	0.3	0.2	0.1	0.01	0.1	0.2	⋯	0.8	0.9	x
1	$Sat_{1}$	0.9	0.8	0.7	0.6	0.5	0.4	0.3	0.2	0.1	0.01	0.1	0.2	⋯	0.8	0.9	x
	$Sat_{2}$	x	0.9	0.8	0.7	0.6	0.5	0.4	0.3	0.2	0.1	0.01	0.1	0.2	⋯	0.8	0.9

## Abbreviations

CDMA	Code Division Multiple Access
CRDSA	Contention Resolution Diversity Slotted Aloha
DtS-IoT	Direct to Satellite IoT
GEO	Geostationary Orbit
HSTN	Hybrid Land-Satellite Network
IoT	Internet of Things
IRSA	Irregular Repetition Slotted Aloha
ITLD	Intelligent Traffic Load Distribution
LEO	Low Earth Orbit
LPWAN	Low Power Wide Area Network
MAC	Media Access Control
MIMO	Multiple Input Multiple Output
RA	Random Access
SAT	Satellite
SIC	Successive Interference Cancellation
TDMA	Time Division Multiple Access
5G	Fifth Generation of Wireless Communication Systems
6G	Sixth Generation of Wireless Communication Systems
∅	Empty set
ε	Erasure probability
$\varepsilon_{k}$	Erasure probability at the *k*th satellite
Δθ	Angular variation
$D_{k}$	Set of data packets successfully received by a different satellite
*G*	Channel load
$G_{T}$	Total traffic load
$G_{i}^{\left(u\right)}$	Uniform traffic load distribution, per position
$G_{i}^{\left(nu\right)}$	Non-uniform traffic load distribution, per position
J	Set with indexes of the subsets whose intersection must be evaluated
*K*	Number of satellites in the constellation
*k*	*k*-th Satellite in orbit
*M*	Positions in each lap
*N*	Number of time-slots
*n*	Number of erasure probabilities
$P_{k}$	Position of the *k*th satellite
P	Probability that users transmit in the same time-slot
P	Packet loss rate
$P_{k}$	Packet loss rate at the *k*th satellite
$p_{J}$	Probability of successful reception, of a given packet, by |J| satellites
$Q_{i}$	Load factor
$q_{J}$	Probability of successful reception of the same packet by by |J| satellites,
	given that *u* packets were transmitted
$q_{k}$	Probability of successful packet reception at the *k*th satellite
$Sat_{1}$	First LEO satellite
$Sat_{2}$	Second LEO satellite
*s*	Satellite spacing
T	System throughput
$T_{i}$	Throughput per position
$T_{k}$	Throughput at the *k*th satellite
$T^{\left(ITLD\right)}$	Throughput for Intelligent Traffic Load Distribution
$T^{\left(nu\right)}$	Throughput for non-uniform distribution
$T^{\left(u\right)}$	Throughput for uniform distribution
*U*	Poisson random variable
*u*	Number of users transmitting in the same time-slot
